# Lidocaine Induces Endoplasmic Reticulum Stress-Associated Apoptosis *in Vitro* and *in Vivo*

**DOI:** 10.3390/ijms12117652

**Published:** 2011-11-08

**Authors:** Dae Young Hong, Kisang Kwon, Kyeong Ryong Lee, Young Jin Choi, Tae-Won Goo, Kweon Yu, Seung-Whan Kim, O-Yu Kwon

**Affiliations:** 1 Department of Emergency Medicine, Konkuk University Medical Center, Seoul 143-729, Korea; E-Mails: sean1011@daum.net (D.Y.H.); lkrer@kuh.ac.kr (K.R.L.); 2 Department of Anatomy, Chungnam National University, Taejon 301-747, Korea; E-Mails: ppkisang@empas.com (K.K.); choiyj@cnu.ac.kr (Y.J.C.); 3 Department of Agricultural Biology, National Academy of Agricultural Science, RDA, Suwon 441-100, Korea; E-Mail: gootw@korea.kr; 4 Korea Research Institute of Bioscience and Biotechnology, Taejon 305-806, Korea; E-Mail: kweonyu@kribb.re.kr; 5 Department of Emergency Medicine, Chungnam National University Hospital, Taejon 301-721, Korea

**Keywords:** lidocaine, endoplasmic reticulum (ER) stress, ER chaperone, ER stress sensor, apoptosis

## Abstract

We demonstrated that upregulation of both gene expression of endoplasmic reticulum (ER) stress chaperones (BiP, calnexin, calreticulin, and PDI) and ER stress sensors (ATF6, IRE1 and PERK) was induced by lidocaine, a local anesthetic, in PC12 cells. In addition to gene regulation, lidocaine also induced typical ER stress phenomena such as ART6 proteolytic cleavage, eIF2 alpha phosphorylation, and XBP1 mRNA splicing. In *in vivo* experiments, while lidocaine downregulated gene expression of antiapoptotic factors (Bcl-2 and Bcl-xl), pro-apoptotic factor (Bak and Bax) gene expression was upregulated. Furthermore, lidocaine induced apoptosis, as measured histochemically, and upregulated PARP1, a DNA damage repair enzyme. These results are the first to show that lidocaine induces apoptosis through ER stress *in vitro* and *in vivo*.

## 1. Introduction

Lidocaine, an amino amide-type of local anesthetic, was first synthesized in 1943. Lidocaine is a local or topical anesthetic that can be applied to the skin or to mucous membranes to reduce the immediate feeling of pain and produce numbness. Due to its rapid onset of action and intermediate efficacy, lidocaine is widely used in both emergency departments and dental offices. Lidocaine acts on the nerve axon sodium channels to prevent depolarization, but the molecular mechanisms are unclear [1]. Lidocaine toxicity occurs following unintended intravascular administration or administration of an excessive dose. When lidocaine is clinically used for regional nerve blocks, plasma levels are usually 3–5 mg/mL. Toxicities may be observed at 6 mg/mL, but more commonly occur once levels exceed 10 mg/mL [2]. The major side effects of lidocaine primarily involve the central nervous system (CNS), the gastrointestinal tract, and the cardiovascular system. Various types of clinical side effects have been reported with lidocaine over-administration in both the CNS (drowsiness, dizziness, apprehension, euphoria, tinnitus) and cardiovascular system (hypotension, bradycardia, cardiovascular collapse). Additionally, it is well known that lidocaine is hepatically metabolized and then finally leads to liver dysfunction. Some reports have demonstrated that lidocaine is significantly toxic to articular chondrocytes [3–7]. The mechanism of lidocaine chondrotoxicity has been intensively investigated, but the molecular mechanisms underlying this cytotoxic outcome are unclear.

Endoplasmic reticulum (ER) is a central organelle of eukaryotic cells and is the location for post-translational modification of secretory and plasma membrane proteins. The ER is also a very important signal transducing organelle and maintains intracellular homeostasis. Conditions interfering with ER function are comprehensively called ER stress. ER stress in the ER lumen is induced by accumulation of unfolded protein aggregates (unfolded protein response, UPR) or by excessive protein production by viral infection (ER overload response, EOR). ER stress has unique signaling pathways to protect cells against UPR and/or EOR involving three main kinds of ER stress sensors (inositol-requiring enzyme 1; IRE1, protein kinase-like ER kinase; PERK, and activating transcription factor 6; ATF6). Under ER stress conditions, BiP (immunoglobulin-binding protein/glucose-regulated protein), binds to unfolded proteins and activates the ER stress response. While activation (autophosphorylation and dimerization) of IRE1 activates the endonuclease domains that cleave X-box DNA-binding protein (XBP) mRNA and generates an activated form of XBP1 by removing 23 nucleotides including a *Pst*I restriction enzyme site, PERK activation results in phosphorylation of the α-subunit of eukaryotic translation initiation factor 2 (eIF2-α) and inhibits translation initiation. ATF6 is cleaved at the cytosolic face of the membrane in response to ER stress, causing nuclear translocation of the *N*-terminal cytoplasmic domain, which contains DNA-binding, dimerization, and transactivation domains, and subsequent binding to both ER stress-response element (ERSE) and ATF6 sites to enhance ER molecular chaperone genes [8–11].

UPR is acutely sensitive to environmental or physical changes and is associated with apoptosis. Proteins downstream of all three UPR pathways involving ER stress sensors have been identified with pro-apoptotic roles. Particularly prolonged and/or strong ER stress promotes apoptosis through the ER signal. However, at present, the mechanism of ER stress-associated apoptosis is not fully understood, because of the degree of cross talk among the pathways. Here, we investigated the effects of lidocaine on ER stress-associated apoptosis in PC12 cells.

## 2. Results and Discussion

UPR is a mechanism by which cells respond to an increase in misfolded or unfolded proteins in the ER lumen. Stressors that evoke the UPR vary but include chemicals, pathogens, environmental factors, and abnormal physiological conditions. Activation of the UPR begins with three ER stress sensors, IRE1-α, PERK, and ATF6, whose lumenal domains act as sensors for malfolded (misfolded and/or unfolded) proteins by competing with them for binding to the ER chaperone BiP [12]. Activation of IRE1-α stimulates cytosolic endoribonuclease to act upon XBP1 mRNA, removing an intron and converting the encoded protein from inactive XBP1 into transcriptionally active XBP1. PERK activates via autophosphorylation, and increases transcription of UPR target genes via eIF2-α phosphorylation. Activation of ATF6 is accomplished in the Golgi complex by two proteases (S1P and S2P), which release a basic leucine zipper transcription factor domain. Acute stress responses are strongly associated with the UPR, the acute responsive cells that undergoes apoptosis via the UPR target CHOP, which inhibits expression of the anti-apoptotic gene Bcl2 and activates pro-apoptotic gene expression [13]. Although lidocaine is a common local anesthetic and an anti-arrhythmic drug, results of some clinical reports show side effects that affect the nerve axon sodium channels and prevent depolarization. However, there is not enough data to understand the side effect at the molecular level. We were interested if the UPR including apoptosis was responsive to lidocaine in cultured cells, because the UPR response depends on changes in the transcription level of particular critical genes.

**Figure 1:**
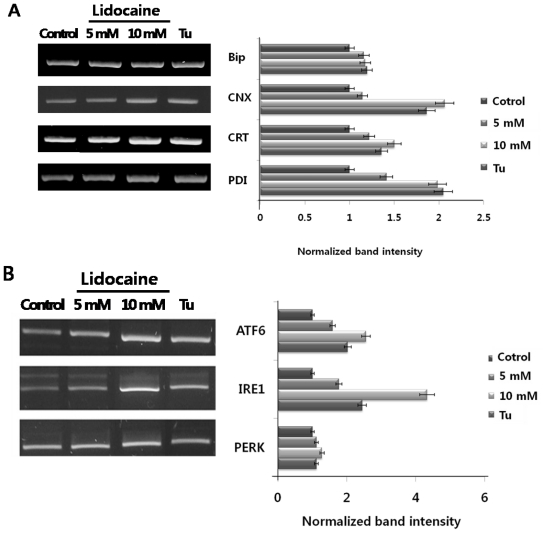
Expression of endoplasmic reticulum (ER) chaperones (**A**) and ER stress sensors (**B**) following lidocaine treatment. PC12 cells were treated with lidocaine at doses of 5 and 10 mM for 12 h. All mRNA levels of immunoglobulin heavy chain binding protein (BiP), calnexin (CNX), calreticulin (CRT), protein disulfide isomerase (PDI), activating transcription factor 6 (ATF6), inositol-requiring 1 (IRE1), protein kinase RNA-like endoplasmic reticulum kinase (PERK) and tunicamycin (Tu) as an ER stress landmark were measured by semiquantitative RT-PCR. All experiments were performed at least three times, and results represent the average. The uptake ratio (fold) is shown relative to the control (1-fold).

So far, no data have shown that lidocaine induces either ER chaperone or ER stress sensor expression. Here, we first tested whether PC12 cells treated with lidocaine induced ER chaperone expression (BiP, calnexin; CNX, calreticulin; CRT, and PDI) and ER stress sensors (ATF6, IRE1, and PERK). The results are shown in (Figure 1A and B). Although the expression of ER luminal chaperone BiP is not remarkable, lidocaine-dose dependently increased CNX, CRT, and PDI mRNA levels. The maximum expression levels (~2 fold) were almost the same as cells treated with the ER stress-inducible drug tunicamycin. PERK expression showed little change, whereas ATF6 and IRE1 expression increased strongly. Maximum expression (ATF6, ~2 fold; IRE1, ~4 fold) levels were higher than cells treated with tunicamycin. These results demonstrate that lidocaine triggers ER stress chaperone and ER stress sensor overexpression and induces ER stress in PC12 cells.

**Figure 2:**
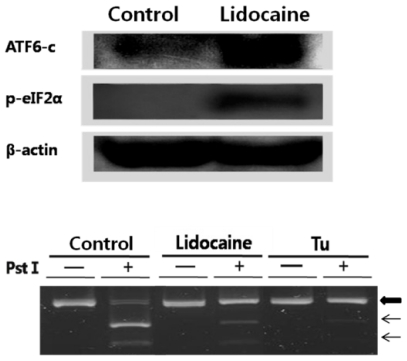
Endoplasmic reticulum (ER) stress transducers including protein kinase RNA-like endoplasmic reticulum kinase (PERK), activating transcription factor 6 (ATF6) and inositol-requiring 1 (IRE1) were activated by lidocaine treatment. ER stress markers including eIF2 alpha phosphorylation (p-eIF2α), ATF6 proteolysis, and X-box binding protein 1 (XBP1) mRNA splicing were examined following ER stress by lidocaine treatment. Upper panel, ATF6 immunoblotting analysis; cells were treated with 10 mM lidocaine for 12 h. Cell lysates were subjected to Western blotting with mouse anti-ATF6 monoclonal antibody and anti-phosphorylated-eIF2α antibody, respectively. Lower panel, after *Pst*I digestion of XBP1 cDNA, the resulting *Pst*I digested XBP1 products were revealed on a 2% agarose gel. Unspliced XBP1 mRNA produced the two lower bands indicated by the small arrows (middle and lower arrow), whereas spliced XBP1 mRNA produced one 450 bp band (upper larger arrow). Although the experiments were performed in triplicate, only a representative blot is shown.

The first ER stress response involving upregulation of genes encoding ER chaperones is demonstrated in Figure 1. The second ER stress response consists of three distinct ER stress sensors that are downstream components of ER chaperones and transmit stress signals from the ER to the nucleus in response to perturbations in protein folding in the ER lumen. To confirm that selected ER stress sensors of the UPR were responsive to lidocaine, we tested their activity. The results are shown in Figure 2. AFT6 was constitutively expressed as a 90 kDa protein. ATF6 was cleaved at the cytosolic face of the membrane in response to ER stress, leading to nuclear translocation of the *N*-terminal cytoplasmic domain, which contained the DNA-binding, dimerization, and transactivation domains. There, the *N*-terminus binds to both ERSE and ATF6 to enhance the ER molecular chaperone genes. We did not observe an increase in total PERK. Although total PERK expression may increase with UPR, the level of phosphorylated PERK is more important to investigate [14]. We used RT-PCR and *Pst*I digestion to confirm the induction of XBP1 mRNA splicing (the proximal step of IRE1) by lidocaine. Both inactive (upper, big arrow) and splice-activated forms (indicated on the figure, lower two arrows) of XBP1 mRNA were recovered. Cells treated with tunicamycin showed strong unspliced XBP1 mRNA indicated by the large arrow, and a similar result was detected in the lidocaine treatment. However, no lidocaine treatment resulted in strongly spliced XBP1 mRNA, indicating that IRE1 kinase activity triggers the attached RNase activity to produce spliced XBP1 mRNA resulting in a cDNA fragment (middle and lower arrows in Figure 2, lower panel). Lidocaine increased eIF2-α expression and phosphorylation (phosphorylation is the proximal step in PERK activation).

**Figure 3:**
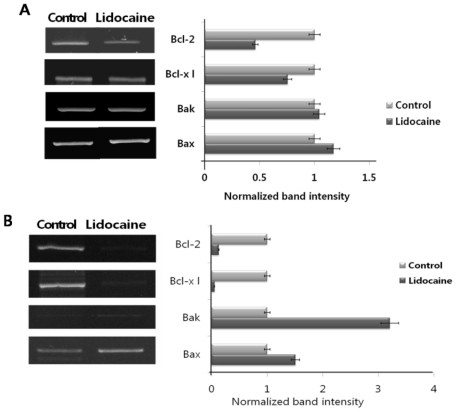
Apoptosis regulator factors are regulated by lidocaine treatment *in vitro* (**A**) and *in vivo* (**B**). PC12 cells were treated with 10 mM lidocaine for 12 h. Rat femurs were treated with 0.5 mL/130 g lidocaine. mRNA levels of B-cell lymphoma 2 (Bcl-2), B-cell lymphoma/leukemia-x long (Bcl-xl), Bcl-2 homologous antagonist/killer (Bak), and BCL2-associated X (Bax) were measured by semiquantitative RT-PCR. All experiments were performed at least three times, and results represent the average. The uptake ratio (fold) is shown relative to the control (1-fold).

Until the results of Figures 1 and 2, the UPR response to lidocaine showed upregulation of ER chaperones and ER stress sensors. The increased availability of these transcriptional regulators altered the expression of many genes, including the core elements of the URP pathway itself, molecular chaperones, proteins involved in the degradation of ER proteins, antioxidant proteins, and anti-apoptotic (Bcl-2 and Bcl-xl) and pro-apoptotic (Bak and Bax) factors. High levels of the Bcl-2 protein protect cells from early apoptotic death by preventing activation of the caspases that carry out the process. Bcl-xl is one of the several anti-apoptotic proteins that are members of the Bcl-2 family of proteins. Bax and Bak play a critical role in the induction of apoptosis. It is known that UPR activates unique pathways that lead to cell death through apoptosis under severe and prolonged ER stress conditions. Furthermore, several pathways have been implicated in ER stress-induced apoptosis. Here, we tested whether UPR induction by lidocaine was associated with apoptosis *in vitro* and *in vivo*. As shown (Figure 3A), cultured cells treated with lidocaine showed downregulation of Bcl-2 and Bcl-xl. However, Bak and Bax expression increased slightly compared to that in the control. The femur was treated with lidocaine, excised, and total RNA was purified. The results of the RT-PCR were basically the same as the cell experiment; however, the result of induction and suppression of apoptosis were more obvious with and without lidocaine treatment (Figure 3B). The *in vitro* and *in vivo* results show that lidocaine stimulation is closely related to apoptosis regulation; downregulation of anti-apoptotic factors such as Bcl-2 and Bcl-xl, and upregulation of pro-apoptotic factors Bak and Bax.

**Figure 4:**
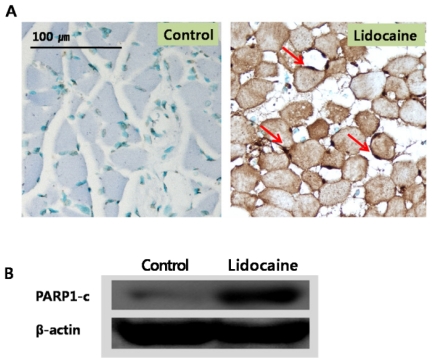
Results of the terminal deoxynucleotidyl transferase-mediated dUTP nick end labeling (TUNEL) assay **(A)** and poly (ADP-ribose) polymerase 1 (PARP1) expression **(B)** in lidocaine-treated skeletal muscle. Lidocaine (0.5 mL) was injected into rat femurs (Sprague–Dawley, average weight 130 g). After 1 day, skeletal muscle was obtained, fixed, embedded, and cut into 4 μm sections. The TUNEL assay was performed using the ApopTag® Plus Peroxidase *in situ* Apoptosis Detection kit. Arrows indicate apoptosis positive signal. For Western blotting, skeletal muscle was homogenized in lysis buffer and then exposed to the mouse anti-PARP1 monoclonal antibody. Although the experiments were performed in triplicate, only a representative blot is shown.

Apoptosis was tested *in vivo* using the terminal deoxynucleotidyl transferase-mediated dUTP nick end labeling (TUNEL) assay and femur tissue treated with lidocaine (Figure 4A and 4B), which is a common method for detecting DNA fragmentation resulting from apoptotic signaling cascades. Compared to the control, myofibers of each tested muscle tissue showed severe DNA damage (indicated by arrows), indicating that lidocaine induced apoptosis as was demonstrated *in vivo*. The poly (ADP-ribose) polymerase 1 (PARP1) protein is involved in various important cellular processes such as differentiation, proliferation, and regulation of the molecular events involved in the recovery of cells from DNA damage [15–17]. Upregulated PARP1 expression by lidocaine was also detected, indicating that lidocaine is involved in the recovery of damaged DNA as well as the induction of apoptosis.

The results of this study give important clues to two aspects of lidocaine use: one is the clinical side by overdose treatment of lidocaine; another is to show the possibility of apoptosis-induction via ER stress.

## 3. Experimental Section

### 3.1. Cell Culture and Lidocaine Treatment

PC12 cells were cultured on collagen coated flasks in 85% RPMI 1640 supplemented with 25 mM Hepes buffer, 10% heat-inactivated horse serum, and 5% heat-inactivated fetal bovine serum, 2 mM l-glutamine, 1 mM sodium pyruvate, 1 g/L d-(+)-glucose, and antibiotics: 25 μg/mL streptomycin and 25 U/mL penicillin. Cells were maintained in a humidified incubator at 37 °C in a 5% CO_2_ atmosphere. The medium was exchanged every 48 h. Cells were rinsed with 1× PBS, pH 7.0, and detached with 0.25% trypsin/EDTA. Following centrifugation (1,000 × *g*, 5 min), cells were sub-cultured in 25-cm^2^ flasks using a sub-cultivation ratio of 1:2 to 1:4 and were photographed every 24 h with an inverted microscope. Cells were passaged twice per week. The 80% confluent PC12 cell monolayer was treated with the indicated doses of lidocaine (S7101, Millipore, Bedford, MA, USA). After another 12 h of culture, total RNA from cultured cells was extracted using an RNA isolation reagent (TRI-Reagent Ambion, Austin, TX, USA) and used for the following RT-PCR experiments.

### 3.2. Semiquantitative RT-PCR and XBP-1 mRNA Splicing

RT-PCR was performed using the forward primer (F) (5′-ACCACCAGTCCATCGCCATT-3′) and reverse primer (R) (5′-CCACCCTGGACGGAAGTTTG-3′) for IRE1, F (5′-AGTGGTGGCCACTAATGGAG-3′) and R (5′-TCTTTTGTCAGG GGTCGTTC-3′) for BiP, F (5′-CTAGGCCTGGAGGCCAGGTT-3′) and R (5′-ACCCTGGAGTATGCGGGTTT-3′) for ATF6, F (5′-GGTCTGGTTCCTTGGTTTCA-3′) and R (5′-TTCGCTGGCTGTGTAACTTG-3′) for PERK, F (5′GGGAGTCTTGTCGTGGAATTG-3′) and R (5′-TGCTTTCCAAGACGGCAGA-3′) for calnexin, F (5′-ACATCAGGAGCTAAAAGCAGCC-3′) and R (5′-TGAAACATACGTCACCCGCA-3′) for calreticulin, F (5′-TAGCAAAGGTGGATGCCACA-3′) and R (5′-CACCATACTGCTGAGCCAGG T-3′) for PDI, F (5′-ACGGCCTTGTGGTTGAGAAC-3′) and R (5′-TGTCCATTCCCAAGCGTGT T-3′) for XBP1, and F (5′-GATCACCATCGGGAATGAACGC-3′) and R (5′-CTTAGAAGCATTTGCGGT GGAC-3′) for α-actin. RT-PCR primers were supplied from Bioneer Co. (Taejon, South Korea). Unless otherwise noted, all other chemicals were purchased from Sigma (St. Louis, MO, USA). RT-PCR conditions were as follows: 30 cycles (94 °C for 30 s, 58 °C for 30 s, and 72 °C for 1 min (with 10 min final cycle) using the above primers with *Taq* DNA polymerase. The splicing status of XBP1 mRNA was detected by RT-PCR and *Pst*I digestion.

### 3.3. Western Blotting

Immunoblotting analysis was performed according to standard procedures. PC12 cells were scraped, lysed by adding SDS sample buffer (62.5 mM Tris. HCl pH 6.8, 6% [w/v] SDS, 30% glycerol, 125 mM DTT, 0.03% [w/v] bromphenol blue), and separated by SDS-PAGE. The proteins were transferred to a nitrocellulose membrane, and the membrane was incubated with primary antibodies overnight at 4 °C. The blots were developed using an Enhanced Chemiluminescence Western Blotting Detection System kit (Amersham, Uppsala, Sweden). Rabbit anti-eIF2 antibody, eIF2-P antibody, and goat anti-actin antibody were obtained from Santa Cruz Biotechnology (Santa Cruz, CA, USA). Mouse anti-ATF6 antibody was obtained from Imgenex (San Diego, CA, USA).

### 3.4. TUNEL Assay

Male Sprague–Dawley rats were kept under a 12 h light-dark cycle with free access to standard rat feed and tap water. Lidocaine (0.5 mL) was injected into the femur of rats (average weight 130 g), and skeletal muscle was obtained after 1 day under phenobarbital anesthesia (50 mg/kg intraperitoneally). The tissues were fixed immediately in buffered 4% paraformaldehyde. One aliquot of muscle was homogenized for Western blotting analysis. Another aliquot was dehydrated and embedded in paraffin wax and cut into 4-μm sections. The TUNEL assay was performed using the ApopTag® Plus Peroxidase *in situ* Apoptosis Detection kit (Millipore). Paraffin-embedded sections were deparaffinized with absolute and 95, 75, and 50% ethanol solutions and then washed with PBS. After endogenous peroxidase was inactivated in 3% hydrogen peroxide, the slide preparations were treated with 50 mg proteinase K per ml for 30 min at room temperature. Thereafter, the sections were incubated for 90 min at 37 °C with terminal deoxyribonucleotidyl transferase (75 U/mL) and 5 mM digoxigenin-11-dUTP in potassium 200 mM cacodylate buffer (pH 8.0) containing bovine serum albumin (50 μg/mL) and 2.5 mM CoCl_2_. After 90 min, the slides were washed with SSC buffer (150 mM NaCl, 15 mM sodium citrate, pH 7.0), followed by 10 mM Tris/HCl (pH 8.2) in 150 mM NaCl. Non-specific binding was blocked with a blocking reagent for 30 min at room temperature. Labeled nick ends of DNA strands were visualized with the alkaline phosphatase reaction. The reaction was stopped after 15 min by washing with H_2_O.

## 4. Conclusions

In summary, lidocaine, a local anesthetic, induced upregulation of ER stress chaperones (BiP, calnexin, calreticulin, and PDI) and ER stress sensors (ATF6 and IRE1) in PC12 cells. In addition to the upregulation of gene expression, lidocaine also induced ART6 proteolytic cleavage, eIF2-α phosphorylation, and XBP1 mRNA splicing. Through *in vitro* and *in vivo* experiments, it was demonstrated that lidocaine was closely related to the regulation of expression of both the anti-apoptotic factors Bcl-2 and Bcl-xl and the pro-apoptotic factors Bak and Bax. Lidocaine also induced apoptosis, as assayed histochemically, and upregulated PARP1, which helps to repair damaged DNA. Based on these results, we suggest that lidocaine induces ER stress-associated apoptosis.
